# Detection and Analysis of Bionic Motion Pose of Single Leg and Hip Joint Based on Random Process

**DOI:** 10.3389/fbioe.2022.884318

**Published:** 2022-04-27

**Authors:** Peng Zhang, Seung-soo Baek

**Affiliations:** ^1^ School of Physical Education, Xinyang Normal University, Xinyang, China; ^2^ School of Physical Education, Sangmyung University, Seoul, Korea

**Keywords:** motion pose detection, dynamic model, stochastic process, single leg and hip joint bionics, flexible swivel hip joint

## Abstract

Based on the spectral representation method of random function and combined with memoryless nonlinear translation theory, this paper analyzes the transformation relationship between potential Gaussian random process and non-Gaussian random process, and successfully generates a stationary non-Gaussian random process that conforms to the target non-Gaussian random process. For the non-stationary non-Gaussian random process simulation, on the basis of the stationary Gaussian random process, the intensity non-stationary uniform modulation model is used to modulate it, and combined with the nonlinear translation theory, the non-stationary non-Gaussian random process conforming to the target non-Gaussian random process is obtained. Aiming at the single-leg bouncing model based on the flexible rotary hip joint, the stability of its bouncing motion under passive motion is studied, and the influence of the flexible hip rotary joint on the motion stability is analyzed by comparing the single-leg bouncing motion characteristics of the free rotary hip joint. Based on the inverse dynamic control of the air phase, the fixed point distribution of the single-leg bounce of the flexible rotary hip joint was improved, and the function of the flexible rotary hip joint in the energy conversion of the bouncing motion was studied by establishing the energy consumption evaluation function. The kinematic performance verification, dynamic performance verification, dynamic parameter identification verification, and modal experiment simulation analysis were carried out for the built experimental platform, and the comparison and analysis with its theoretical model were carried out. The results show that the theoretical motion trajectory of the test mobile platform is basically consistent with the actual motion trajectory in the X and Y directions, and there is a small error in the Z-axis direction, and the error is within an acceptable range, indicating that the experimental platform system can be used to simulate the human hip joint. There is a large error between the theoretical value of the driving torque calculated by the theoretical value of the dynamic parameters and the measured value, and the dynamic theoretical model cannot accurately predict the driving torque. The predicted value of the driving torque calculated by using the identification value of the dynamic parameters is in good agreement with the measured torque, and its confidence is increased by 10–16%, indicating that the dynamic parameter identification method in this paper has a high degree of confidence.

## Introduction

In recent years, due to the development of science and technology and the expansion of human activity space, the research on mobile robots has received great attention, and there are more and more types of mobile robots, ranging from entertainment robot toys, household service robots, to engineering adventures (Yang et al., 2019). Mobile robots can move to the goals set by people and complete the set operation tasks. Correspondingly, the practicability of mobile robots has higher and higher requirements for mobile robot systems in terms of moving speed, flexibility, autonomy, and work ability. Existing mobile robots are generally footed or wheeled robots. The footed robot can walk stably in a complex environment, cross large trenches and steps, and move flexibly (Leng et al., 2021). However, the level of the existing mechanism leads to a generally low speed of movement; the wheeled robot is relatively easy to drive and control, has a high speed of movement on flat ground, is responsive, and has high stability (Chen et al., 2021a; Zheng et al., 2021; Chen et al., 2022a). However, the obstacle avoidance ability of legged robots and wheeled robots is not good enough, and their mobility in complex terrain also needs to be improved.

According to the principle of bionics and the requirements of human body structure and motion characteristics, using the parallel mechanism as the joint configuration of the humanoid robot is in line with the objective reality and is an important research direction to realize the bionic body of the humanoid robot (Hasan and Dhingra, 2020). The hip joint helps the human body stand, and realizes flexion and extension, internal rotation and external rotation, adduction and abduction, that is, the three-dimensional rotation of the human lower limb around the geometric center of the femoral socket, and plays an irreplaceable role in various movements (Leng et al., 2020). It is one of the most important joints in the human body (Liu et al., 2019). Combining all kinds of parallel mechanisms, the spherical parallel mechanism with few degrees of freedom is more in line with the characteristics of the human hip joint in structure and function, so it is necessary to combine the characteristics of the human hip joint to apply the spherical parallel mechanism with few degrees of freedom in the hip joint application of humanoid robots (Zhang et al., 2021; Liu X. et al., 2022).

The numerical method based on the Mehler formula solves the equivalent correlation coefficient and realizes the simulation of non-stationary and non-Gaussian random processes (Zheng et al., 2022). The core work of the non-Gaussian stochastic process simulation method based on memoryless nonlinear translation lies in the transformation of a potential Gaussian stochastic process conforming to the target non-Gaussian stochastic process to a non-Gaussian stochastic process, and the focus is on the transformation of the correlation coefficient between the two (Wang G. et al., 2021; Yun et al., 2022). The traditional calculation method of correlation coefficient transformation involves solving complex two-dimensional integral problems (Li F. et al., 2020; Chen et al., 2022b). This paper solves the conversion problem of equivalent correlation coefficient in non-Gaussian process simulation based on the Mehler formula, and combines the random function-spectral representation method to realize the simulation of non-Gaussian random process efficiently and accurately. In this paper, a simplified dynamic model of single-leg bouncing that can describe the flexible rotation characteristics of the hip joint is established, and the dynamic equations of its flight phase and landing phase are obtained, and the periodic motion characteristics are analyzed by Poincaré mapping. On this basis, a fixed point search strategy based on the rough search of periodic cell map and the precise search of orthogonal table is studied to solve the problem of search calculation complexity caused by the large parameter dimension of the flexible rotary joint bouncing model. According to the topology diagram of the parallel mechanism and the experimental standard of artificial hip joint friction and wear, the mechanical system, electrical system and software system of the testing machine are designed, and the experimental platform system of the parallel bionic hip joint testing machine is built. The relevant experimental data are collected by sensors such as instrument, and the kinematic performance, dynamic performance and dynamic parameter identification results of the built experimental platform are verified and analyzed. Modal experiments were carried out on the experimental platform built by Donghua test system, acceleration sensor, force hammer and other instruments. The error of the natural frequency calculated by the elastic dynamic model and the first second-order natural frequency of the testing machine obtained by the modal test experiment are all within 8%, and the error is within the acceptable range, which verifies the elastic dynamic model.

## Related Work

At present, the representative products of the common bionic legs on the foreign market are the Cheetah series, Flex-foot series and Total Knee series designed by Iceland’s OSSUR company (Sharifi and Shirazi-Adl, 2021; Wu et al., 2022). The bionic legs of the Cheetah series imitate the foot structure of a cheetah so that it has the effect of energy feedback during exercise, improving the balance and stability of the user’s movement, and the user can participate in various sports. The Total Knee series adjusts the swing motion of the bionic leg through the hydraulic system, so that it has a certain adaptability to the change of pace (Jiang et al., 2021a). However, both passive and semi-active bionic legs cannot provide the user with active torque to meet the requirements of the user’s moderate energy consumption. In the normal human body, the muscles of the legs will provide active torque to generate thrust and reduce the impact when landing (Chiu et al., 2021). Therefore, passive bionic legs will cause more consumption to the user and are not suitable for road conditions such as slopes and stairs.

Biped robots and quadruped robots mainly imitate the leg structure of humans and other mammals. The main joints are distributed in the vertical plane, and the whole is an upright leg structure. This kind of robot travels in a similar way to mammals, and can perform complex movements such as running and jumping. It has a strong ability to overcome obstacles, has a variety of ways to overcome obstacles, and can be used with a robotic arm to achieve construction operations in a wide range. Although the performance is excellent, it is not suitable for large-scale carrying transportation because of the poor stability of the whole machine and the high processing difficulty (Jiang et al., 2021b). Relatively speaking, the hexapod robot mainly imitates the leg structure of crawling insects, and its joints are distributed in the vertical plane and the horizontal plane (Chen et al., 2021b). This kind of robot occupies a relatively large area, and its movement flexibility is not as good as that of quadruped and biped robots. However, because of its high stability and strong carrying capacity, it is generally used in the field of large-scale carrying transportation.

The bionic magnetic control knee joint Rheo Knee series designed by Ossur in Iceland has good self-adaptability, can control the support period and swing period in real time, and can enter the oscillator stably under different road conditions. And through intelligent algorithms, it expands the movement ability of squatting and standing up and improves the function of crossing obstacles and walking backwards (Jiang et al., 2019a). The Linx designed by the British ENDOLITE company provides users with a comfortable experience that approximates the movement of human legs by actively sensing and analyzing the user’s lower limb movement, environment and terrain data, thereby providing a stable and coordinated control command flow for the system’s support system (Yan et al., 2020). By analyzing the user’s exercise data, it provides the patient with the least energy consumption, reduces the probability of arthritis in patients with lower extremity amputation, and reduces the user’s risk of falling and falling through different levels of support, improving the stability of the patient’s movement (Zhou et al., 2021).

Although there are differences in research content at home and abroad, the instruments and methods used for gait testing are almost the same (Li F. et al., 2020). Photography or video equipment is used to record and calculate the motion parameters that need to be measured, and force plates are used to record power. Some research institutions use the plantar pressure distributor to measure the more accurate pressure distribution between the foot and the ground, and also use the electromyography to collect the electromyographic signals of the relevant muscles, etc., and finally conduct a comprehensive analysis (Li T. et al., 2020; Liu et al., 2022a; Chen et al., 2022c).

In order to solve the problem of parameter configuration of bionic legs in clinical promotion, Professor Jennie Si from Arizona State University applied adaptive dynamic programming to the stability control of dynamic bionic legs, using direct heuristic dynamic programming (dHDP) method in two hundred steps (Wang X. et al., 2021; Liu et al., 2022b). The controller parameter configuration of the finite state machine is completed within the framework, and the validity of the method is verified in the opensim simulation. In order to reduce the configuration time of the controller, an improved internal control strategy is proposed (Liu et al., 2021). Related scholars configure the impedance control parameters of the controller through a set of heuristic control parameters and a set of motion state values for normal motion, and the effect is significantly shortened (Park et al., 2020). In addition, with the development of related disciplines such as pattern recognition, there are more and more studies on the control of dynamic bionic legs by using human EMG signals as control signals (Gogoi et al., 2021). The combination of the signal and the neuromuscular reflex model framework can be directly used for the control of the dynamic prosthesis to realize the stable switching of the prosthesis on the level ground and between up and down stairs. Using continuous proportional EMG control, the amplitude of the EMG signal collected in the residual limb is proportional to the prosthetic joint torque or power, allowing the wearer to adjust the ankle prosthetic torque throughout the gait cycle (Kang et al., 2019).

## Methods

### Non-stationary Non-Gaussian Random Process

The first-order probability distribution function and first-order probability density function of a random process X(t) are defined as:

$$F_{X}\left(x,t\right)=P\left\{-x<X\left(t\right)<x\right\}$$ (1)

$$f_{X}\left(x,-t\right)=\partial F_{X}\left(x,-t\right)/\partial x$$ (2)

Likewise, a probability distribution function of order n and a probability density function of order n can be defined for a stochastic process *X(t)*. It is difficult to obtain the distribution function and probability density function of random process in actual situations. Therefore, we consider numerical features that introduce stochastic processes.

The mathematical expectation of a stochastic process is the statistical average of the stochastic process at time *t*, a deterministic function of time, defined as:

$$u_{X}\left(t\right)=\int_{0}^{+\infty }\left|t\right|•f_{X}\left(x,-t\right)dx$$ (3)

It is generally related to *t*, and *X(t)* is called the mean function of the random process.

For a stationary Gaussian random process *X(t)* whose power spectral density is *SX(ω)*, there are two mutually orthogonal real processes *μ(ω)* and *ν(ω)* whose increments d*μ(ω)* and d*ν(ω)* are orthogonal to each other, then there is:

$$X\left(t\right)=\int_{-\infty }^{+\infty }\left\{\sin\left(wt\right)du\left(w\right)-2\cos\left(wt\right)dv\left(w\right)\right\}$$ (4)

The increments d*μ(ω)* and d*ν(ω)* of the processes *μ(ω)* and *ν(ω)* satisfy the following conditions:

$$E\left[du^{2}\left(w\right)\right]-0.5E\left[dv^{2}\left(w\right)\right]=S_{x}\left(w\right)dw$$ (5)

Discrete, we can get:

$$X\left(t\right)=\prod_{k=-1}^{N-1}\left\{\sin\left(w_{k}t\right)\Delta u\left(w_{k}\right)-0.5\Delta k•\cos\left(w_{k}t\right)\Delta u\left(w_{k}\right)\right\}$$ (6)

In the formula, *ω*_*k*_ = *kΔω*, and *Δω* should be small enough.

The number of random variables required by the random function-spectral representation method is different according to its structural form (Huang et al., 2021; Sun et al., 2022). Only 1–2 basic random variables can be used to realize the random process simulation based on the spectral method, which is a good solution to the computational complexity of the spectral method. Problems involving a large number of random variables provide an important basis for efficient analysis of stochastic process fitting and stochastic dynamic response and reliability problems in complex engineering.

An important method for non-Gaussian random process simulation is the nonlinear translation method. The translation process is mainly the mapping process of the edge probability distribution functions of two types of random processes, namely:

$$Y\left(t\right)=T\left[X\left(t\right)\right]•F_{G}\left\{F_{NG}^{-1}\left[X\left(-t\right)\right]\right\}$$ (7)

In the formula, *F*_*G*_ ***(•)*** and *F*_*NG*_ ***(•)*** represent the standard Gaussian edge probability distribution function and the specified non-Gaussian edge probability distribution function, respectively; *F*_*NG*_^*−1*^ ***(•)*** is the inverse function of *F_**NG**_ **(•)***; ***T(•)*** is process transformation function.

### Dynamic Model of Single-Leg Bounce

A single-legged bouncing robot usually consists of a body carrying power and related loads, and a single leg hinged with the body. Corresponding to the structure of animal legs, the joint connecting the legs to the body is the hip joint. According to the single-legged robot’s bouncing motion located in a three-dimensional space or a two-dimensional plane, it can be classified into two categories: three-dimensional bouncing and two-dimensional bouncing. This paper aims to study the influence of hip joint rotational flexibility on the two-dimensional bouncing motion characteristics of the robot, so the hip joint only needs to provide rotational degrees of freedom in the sagittal plane. In order to realize the bouncing motion, the legs of the robot usually have spring components to realize the recycling of energy during the motion. In this paper, in order to study the effect of hip joint flexibility in the single-leg bouncing motion, a torsion spring is set at the position of the hip joint.

In passive motion, the hip joint motor only maintains the current position, and the movement of the hip joint is only provided by the torsion spring connected in series with the motor. Assuming that there is no damping of the spring in the model, the center of mass of the system is located at the center of rotation of the hip joint, and when the robot bounces to the ground, there is no relative sliding between the foot end and the ground (Jiang et al., 2019b). The system bounces to keep the mechanical energy conserved, assuming that the mass of the leg is zero, so there is no energy loss during the landing process, and the energy remains constant throughout the process. This assumption is often used by scholars to analyze simplified models of single-leg bounce.

The motion of the single-legged robot is restricted to bouncing in a vertical two-dimensional plane. Among them, for the landing phase of the bounce, the foot end is in contact with the ground, and *y* = *rcosθ* is used as the judgment condition; the vacant phase is separated from the ground, and *y > rcosθ* is used as the judgment condition. It is assumed that the model is a conservative system in motion, that is, the external energy input to the system during its motion is zero, there is no damping and energy loss caused by collision in the motion of the system, and the total energy loss of the system is also zero. The dynamic equations of each bouncing motion state phase are uniformly expressed as:

$$1-0.5\frac{\partial L}{\partial q}-\frac{d}{dt}\left(\frac{\partial L}{\partial \overset{•}{q}}\right)=0$$ (8)

In the landing phase of bouncing motion, the establishment of polar coordinates is convenient for dynamic analysis. We select *q* as the generalized coordinate, the zero potential energy surface is selected as the ground in the system model, and the Lagrangian function *L* is:

$$L=0.5k_{t}{\left(r-r_{0}\right)}^{2}-0.5k_{h}{\left(\theta -\varphi \right)}^{2}-Mgr\sin\theta$$ (9)

During the bounce-to-ground phase, there are various state variables in the system, such as the body pitch angle and leg swing angle, and there is a coupling relationship between the state variables. The system exhibits obvious nonlinear characteristics. The analytical solution is very difficult, so the numerical simulation method can be used to simplify the solution process, and good analysis results can be obtained by selecting an appropriate simulation step size.

In the bounce phase, the robot is out of contact with the ground. At this time, because the system is a conservative system, it is only affected by gravity, and its center of gravity is a parabolic motion and a uniform motion in the vertical and horizontal directions, respectively. For this case, it can be analyzed in a Cartesian coordinate system. The single-leg system is only affected by the vertical gravity in the air phase, so the trajectory of the robot’s center of gravity is a parabolic motion.

### Dynamics-Based Joint Torque Calculation

**FIGURE 1:**
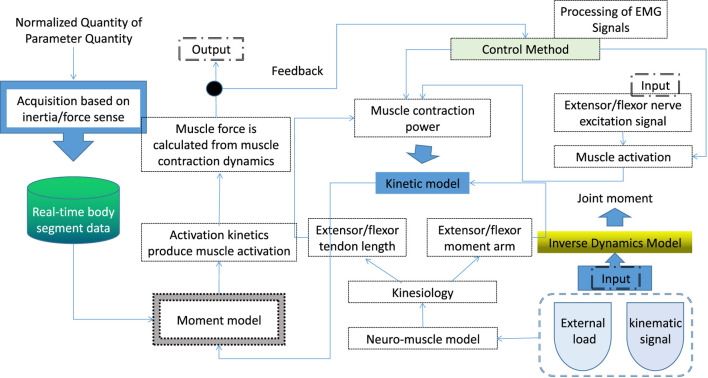
Logic diagram of joint torque calculation.

As shown in Figure 1, the joint torque calculation method consists of four parts: musculoskeletal model, electromyographic torque model, inverse dynamics and parameter optimization algorithm. The EMG torque model covers the process from the nerve stimulation of the muscle to the generation of muscle tendon force and the resulting joint torque; the purpose of the inverse dynamics modeling is to estimate the joint reference torque based on the motion data and external loads. The algorithm determines a specific set of patient parameters that guarantee good joint torque prediction. The human musculoskeletal model is first provided with muscle kinematic data such as muscle-tendon length and moment arm of muscle torque. Muscle kinematic data were used for extensor and flexor contraction kinetic models and kinetic models. Neural stimulation of the extensor and flexor muscle groups is represented by rectified raw EMG signals, resulting in muscle activation through activation kinetics. On this basis, the dynamic method is used to study the joint torque.

### Fixed Point Search Method for Single-Leg Passive Bounce of Flexible Rotary Hip Joint

The single-leg bouncing model of the free-rotating hip joint can be simplified as a SLIP model for analysis. The dynamic equation of the Poincaré interface system has only two state variables, so only two-dimensional search can be performed to obtain the distribution characteristics of its fixed points. However, for the single-leg bouncing model of the flexible rotary joint, its dynamic equation is much more complicated and contains more state variables, so other methods need to be used to study its fixed point.

The cell mapping search method divides the state variables at the Poincaré section in adjacent change domains, each division domain becomes a cell, and the center of each cell is used as a mapping point for block search. This method is used in this paper to outline search.

If a cell can be mapped to itself, the cell is an attracting cell, and its center point exists as a stable fixed point; if a cell near a cell can be mapped to an attracting cell, it is defined as a periodic cell. The set of periodic cells is defined as the domain of attraction of fixed points.

We set several representative spring stiffness ratios, calculate and compare the number of attractive cells obtained from the search, and the results are shown in Table 1. It can be seen that when the stiffness ratio conforms to the above derivation results, there are attractive cells.

**TABLE 1:** The number of attractive cells obtained by searching under typical stiffness ratios.

(k_h_, k_t_)	Number of attracting cells	(k_h_, k_t_)	Number of attracting cells	(k_h_, k_t_)	Number of attracting cells
(0.6, 2.1e4)	2	(4.6, 3.1e4)	1	(5, 2.5e4)	1
(2.6, 1.7e4)	0	(0.8, 2.5e4)	4	(0.3, 2.2e4)	0
(0.7, 2.5e4)	3	(4, 3.1e4)	2	(7.3, 1.4e4)	2

### Single-Leg Bounce Control Method of Flexible Rotary Hip Joint

In this paper, the hip joint drive adopts the motor series elastic drive, and the output torque of the motor is the introduced control variables *τ*_*1*_ and *τ*_*2*_. *M* is the motor fixedly connected to the body, *θ*_*1*_ is the rotation angle of the motor shaft, *θ* is the rotation angle of the leg, and the rotation flexible output characteristic of the hip joint is realized by a series torsion spring between the motor and the leg, and the hip joint torsion is not considered here. Its stiffness is a constant value set theoretically.

The kinetic equation can be:

$$\left\{ \begin{array}{l} \tau_{1}=k_{t}•\left(\theta -\theta_{1}\right)\\ \tau_{2}=k_{h}•\cos\left(\theta -\theta_{1}\right)\\ J_{b}\varphi =\left|\tau_{1}-\tau_{2}\right| \end{array}\right.$$ (10)

Under the stable bouncing motion, the symmetrical flying trajectory corresponds to the symmetrical torque input, *τ*_1_ +*τ*_2_ = 0, this control method is defined as the flying phase control method based on inverse dynamics, and the torque input of the two control phases before and after this control algorithm is used. By doing positive work and negative work respectively, the energy of the system can still maintain the original level during the whole bounce cycle without being affected.

In the landing phase, the bouncing model is in a passive state. In order to be close to the actual application, the collision energy loss is considered. At the same time, due to the input of the control torque in the system, the bouncing process is not a purely passive process.

In the lift-off event, there is no energy loss, and the SLIP model of the state switching process is the same. So far, this section has established the leg control method of the bouncing model with flexible rotary joints in the air phase, and given the state switching during the landing process, so that it can be dynamically analyzed.

## Simulation Experiment and Result Analysis

### Kinematic Performance Simulation

**FIGURE 2:**
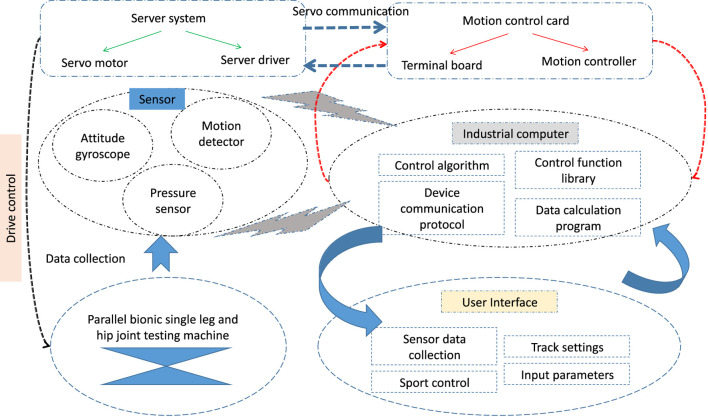
System framework of the experimental platform.

In this paper, the experimental platform system of the parallel bionic hip joint testing machine is built, and the system framework of the experimental platform system is shown in Figure 2. The system is mainly composed of fixed platform, linear module, connecting rod, Hook hinge, ball pair, moving platform components and hydraulic loading system, among which the linear module, Hook hinge, ball pair and hydraulic loading system are purchased parts, and the rest of the components are completed by self-machined processing. Through the connection of the mechanical structure, the rotation of the linear module servo motor is used to drive the test mobile platform to move according to a certain law.

The parallel bionic hip joint testing machine has three degrees of freedom. The servo motor in the linear module drives the ball screw to rotate, and then drives the slider installed on the ball screw to reciprocate up and down. The sliding block drives the moving platform to rotate in three degrees of freedom through the connecting rod. The hydraulic loading system is mainly composed of hydraulic pump, proportional valve, reversing valve and oil cylinder (Zhao et al., 2022). The dynamic loading force is applied to the artificial hip joint experimental material on the moving platform by controlling the opening size of the proportional valve.

The software system is mainly used to complete the task of human-computer interaction, realize the communication function between the various devices of the experimental platform, and process and analyze the data. The control software of this experimental platform is developed under the Windows environment through Visual Basic software according to the requirements of humanization, systematization and functionalization. It also has functions such as collection, analysis and display, and also has the ability to save the collected data into a txt file and store it in the hard disk.

**FIGURE 3:**
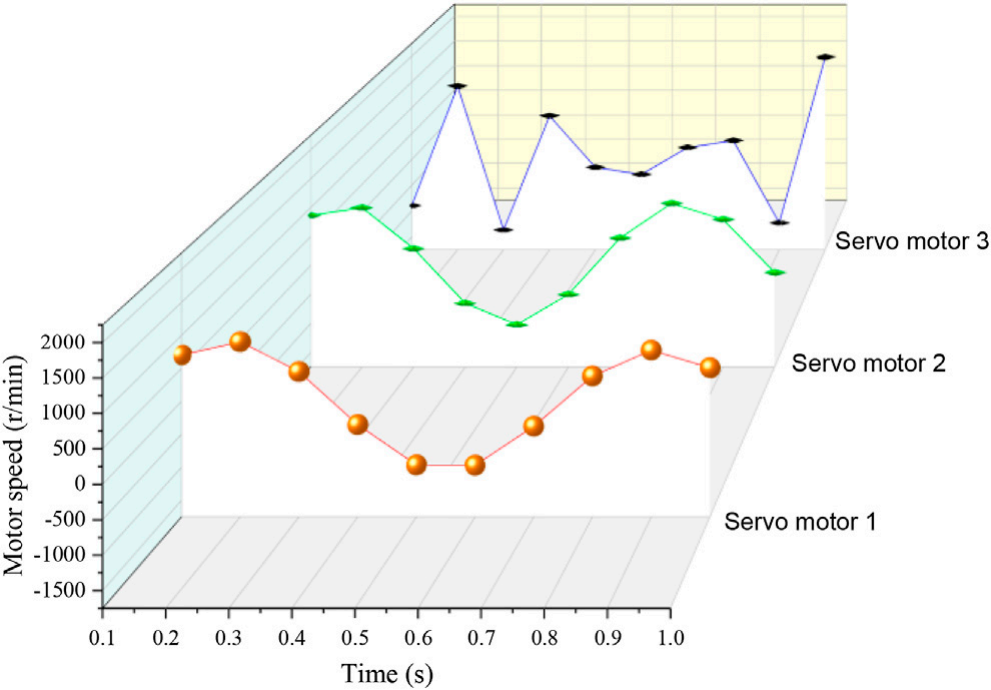
Rotation curve of servo motor.

Using the kinematics model, the speed variation curves of three groups of linear module servo motors can be solved, and their speeds are shown in Figure 3.

**FIGURE 4:**
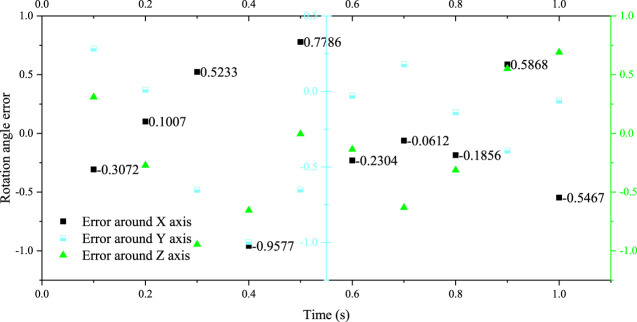
Analysis of the angle error of the moving platform.

Taking the servo motor speed data shown in Figure 3 as the input of the testing machine system, the relevant trajectory parameters are set in the upper computer software system, and the testing machine is controlled to move according to the specified trajectory. In order to improve the accuracy of the experimental data, the experimental platform is made to perform multi-cycle continuous motion, the attitude gyroscope is used to measure the position and attitude of the moving platform in real time, the data in the middle period is selected as the experimental data, and it is compared and analyzed with the preset motion trajectory of the moving platform. The results are shown in Figure 4.

The theoretical motion angle of the moving platform is basically the same as the actual motion angle in the X and Y axis directions, and the angle error is within 0.3°. The error in the Z-axis direction is relatively large, the rotation angle error is about 0.5°, and the maximum error is 1.6°. Compared with the range of the rotation angle of the moving platform around the Z-axis, the error value is within the acceptable range. Therefore, it shows that the inverse kinematics model established is correct, and the experimental platform system built can simulate the motion law of the hip joint under the ISO standard.

Through the comparative analysis of the error between the experimental data and the theoretical data, it can be seen that the rotation angle error of the moving platform around the X and Y directions is basically kept within 0.3°, and the error is relatively small; the rotation angle error of the moving platform around the Z-axis direction is relatively large, and the maximum error is 1.6°, and the larger error mainly occurs in the time period of 0 ∼ 0.6 s. Combined with the loading situation of the testing machine, it can be seen that the loading state of the loading system during this period has a great impact on the motion trajectory of the testing machine.

### Dynamic Performance Simulation

**FIGURE 5:**
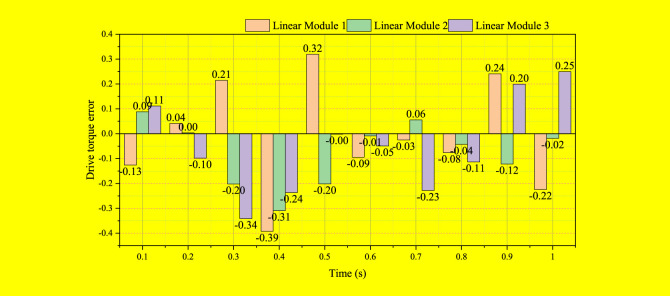
Driving torque error of linear module of testing machine.

On the basis of the kinematics performance verification, at the same time, the servo driver torque monitoring function is used to measure the driving torque of the three groups of linear modules during the movement of the testing machine, and compare and analyze with the theoretical value of the driving torque, as shown in Figure 5.

It can be seen from Figure 5 that the theoretical and measured values of the driving torque of the three groups of linear modules have a relatively large error in the time period of 0 ∼ 0.6 s, and the error value is between 0.05 N m and 0.22 N m. Therefore, the driving torque calculated by the dynamic model cannot predict the true torque of the testing machine.

The calculation results of the theoretical value of the driving torque mainly include two aspects, one is the accuracy of the dynamic model, and the other is the accuracy of the dynamic parameters. The driving torque calculated by the dynamic model has a high fit with the Adams simulation results, and the error is small, so the established dynamic model is correct; the dynamic model solves the dynamic parameters required in the driving torque (friction factor, Inertia tensor, etc.) are the theoretical value or empirical value, and there is a certain error between its value and the actual value. It can be seen that the driving torque error shown in Figure 5 is mainly caused by inaccurate dynamic parameters. Therefore, in order to improve the accuracy of driving torque prediction, it is necessary to identify the actual values of dynamic parameters.

**FIGURE 6:**
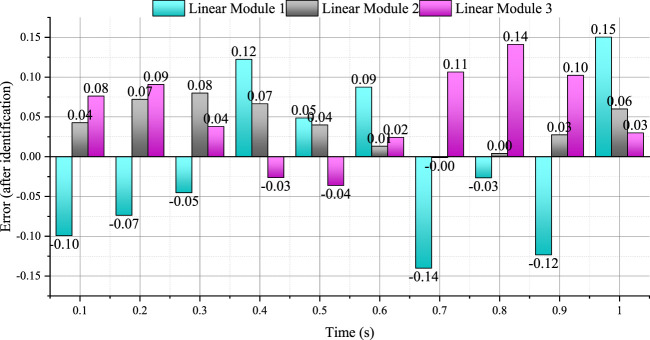
The error between the calculated value and the measured value of the driving torque of the linear module (after identification).

Taking the motion trajectory and the loading force curve as the system input, numerical simulation analysis is carried out, and the calculated value *τ*_*t*_ of the driving torque of the linear module in one motion cycle is obtained. Under the same motion trajectory and loading force, the experimental platform system is used to measure the measured value *τ*_*ci*_ of the driving torque of the linear module in one motion cycle. The error comparison between the calculated value *τ*_*t*_ of the driving torque and the measured value *τ*_*ci*_ is carried out, and the results are shown in Figure 6.

It can be seen from Figure 6 that the errors between the theoretical calculation values of the three groups of linear module driving torques and the experimentally measured values are mainly concentrated in the range of −0.12 N m ∼ 0.15 N m. It can be seen that the calculated value of the driving torque after the identification of the dynamic parameters can well predict the driving torque in the actual situation. Among them, the time period when the driving torque error is large is mainly distributed between 0.4 and 1 s. Combined with the motion trajectory of the moving platform and the loading force curve, it can be seen that the moving platform is in the state of turning around the Z-axis during this time period. Dynamic factors other than the physical parameters are stimulated, resulting in relatively large errors, which need to be further identified and analyzed by other methods.

**FIGURE 7:**
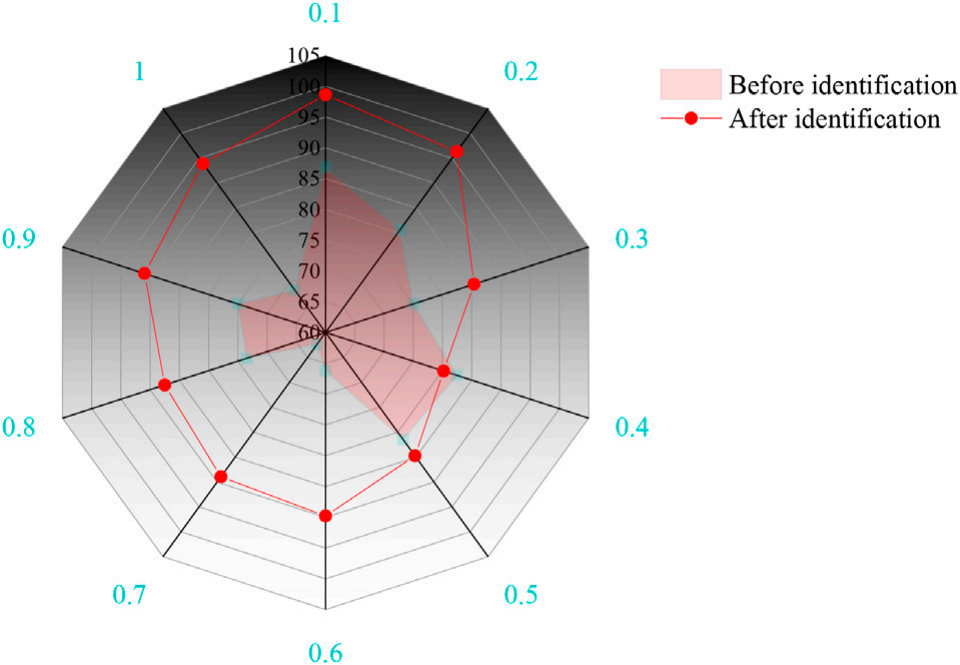
Driving torque confidence.

The confidence of the driving torque is obtained by the dynamic model calculation before and after the identification, and the calculated value *τ*_*t*_ and the measured value *τ*_*ci*_ of the linear module driving torque are further analyzed by using the dynamic parameter identification evaluation index, and the results are shown in Figure 7.

It can be seen from Figure 7 that the confidence of the estimated values of the driving torque of the three groups of linear modules before parameter identification is between 60 and 85%. The confidence of the estimated value of the driving torque calculated from the identification value of the dynamic parameters is between 95 and 100%, which has a high degree of confidence, indicating that the identification results of the dynamic parameters have high reliability and can be used as the driving torque. The confidence of the estimated driving torque calculated by the dynamic parameter identification is more than 20% higher than that of the estimated driving torque before identification.

The time period when the driving torque error is large is mainly distributed between 0.4 and 1 s. During this time period, the moving platform is in the state of turning around the Z-axis. At this time, the dynamic influencing factors other than the above dynamic parameters are stimulated, resulting in a certain error between the predicted value of the driving force and the measured value. This requires further analysis and identification of other parameters using other methods.

### Modal Experiment and Analysis

In this paper, the pulse excitation method is used to conduct the modal experiment, and the hammering method is used as the excitation input of the experimental platform. This experimental method requires less equipment, is simple, convenient and efficient, and is more suitable for modal analysis of the experimental platform of the testing machine.

In order to measure the excitation force at the excitation point, the LC-2 type hammer from Shanghai Zhurui Automation Technology Co., Ltd. was selected for this modal experiment. The sensor of this type of hammer is YDL-4X piezoelectric quartz force sensor. The DHF-7 quartz hammer charge amplifier converts, processes and amplifies the collected force signal, and transmits the signal to the Donghua tester. The hammer has four types of hammer heads, namely steel, aluminum, nylon, and rubber. According to the requirements of this experiment, aluminum hammer heads are selected.

In order to measure the acceleration signal at the measuring point, the 608A11 type ICP single-axis acceleration sensor and 605B31 type ICP three-axis acceleration sensor of PCB company were selected for this modal experiment, and they were fixed at the vibration measuring point by super glue and magnet. The main technical indicators of the equipment are shown in Table 2.

**TABLE 2:** Main technical indicators of the equipment.

608A11/605B31	Sensitivity	100 mv/m.s^−2^
	Frequency Range	0.7Hz–∼10 KHz
	Range	± 50 m.s^−2^
YDL-4X	Sensitivity	4.45 pc/m.s^−2^
	Measuring range	0∼16 kN
	Resolution	0.021 N
DHF-7	Sensitivity	0.81 mv/N
	Working frequency	1 Hz–∼100 KHz
	Maximum charge	10^5^Pc

In the modal analysis, the layout of the measuring points should follow the following principles: more measuring points should be set in the key parts of the structure, and the measuring points in the secondary parts should be sparsely arranged; comprehensive consideration should be given to the location of the maximum deformation of the structure and the nodal line of vibration. Therefore, the measuring points of this experiment are arranged as follows: one measuring point is arranged at each of the three ball hinge installation centers on the moving platform, one measuring point is arranged at the top of the linear module, and one measuring point is arranged at each of the upper and lower sliders. Therefore, the total number of side points that need to be arranged in the testing machine system is 15. Among them, each measuring point needs to measure the acceleration response in three directions of x, y, and z.

In order to analyze the natural frequency of the hip joint testing machine, the vibration is stimulated by hammer tapping, and the test method of single-point excitation input and multi-point acquisition output is adopted. We use the acceleration sensor installed on the linear module and the moving platform to measure the acceleration response signal at the vibration measuring point, and use the Donghua tester to collect and process the relevant signal data. Then, we use Donghua’s own test and analysis software to process and analyze the signal, and the natural frequency of the hip joint testing machine can be obtained.

In the experimental modal module of the DHDAS software, the data type measured in the experiment is defined as force measurement method, the acquisition method is single-point excitation, and the Ployscf algorithm is selected. The Ployscf algorithm is a modal analysis method based on a transfer function. It collects the data of each node through calculation and analysis, and calculates the steady-state distribution diagram of the system. For the data collected in the case of dense system modes or severe noise pollution, the analysis method can still establish a clear system steady state diagram, and has a high recognition accuracy for the parameters of each mode. The steady state diagram is obtained by the Ployscf algorithm, and the natural frequency and mode shape are then analyzed and calculated.

**FIGURE 8:**
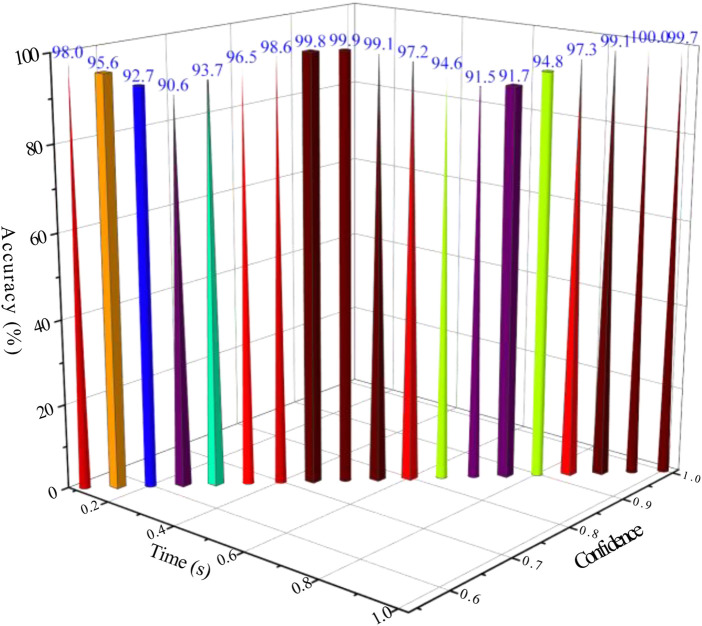
Modal verification.

The modal analysis results obtained by the DHDAS software analysis are modally verified, and the modal MAC analysis results are shown in Figure 8. It can be seen from the figure that the results of the modal parameters are correct.

The DHDAS software is used to carry out modal calculation and analysis on the measured data, and the first second-order natural frequency of the testing machine under a specific pose is obtained. The results are shown in Table 3.

**TABLE 3:** Typical pose natural frequencies.

Pose 1 (2.5°, −6°, 18°)	Upper Moving platform excitation—first order	Upper Moving platform excitation—second order	Lower platform excitation—first order	Lower platform excitation—second order
Simulation calculation value—Hz	62.12	159.30	62.54	157.41
Experimental test value-Hz	71.41	178.23	69.81	176.32
Pose 2 (0.5°, 0°, 2°)	Upper Moving Platform Excitation—First Order	Upper Moving Platform Excitation—Second Order	Lower platform excitation—first order	Lower Platform Excitation—Second Order
Simulation calculation value—Hz	59.91	76.53	59.30	78.52
Experimental test value-Hz	64.31	83.71	63.92	84.61
Pose 3 (8°, −9°, 12°)	Upper Moving Platform Excitation—First Order	Upper Moving Platform Excitation—Second Order	Lower platform excitation—first order	Lower Platform Excitation—Second Order
Simulation calculation value—Hz	10.81	92.14	10.62	91.92
Experimental test value-Hz	12.43	104.72	12.21	102.81

It can be seen from Table 3 that the error of the natural frequency calculated by the elastic dynamic model and the first second-order natural frequency of the testing machine obtained by the modal test experiment are both within 13%, and the error is within the acceptable range. The natural frequency of the system calculated by the simulation can reflect the actual natural frequency of the testing machine system to a certain extent, which verifies the accuracy of the elastic dynamic model.

From the analysis of the natural frequencies of pose 1, pose 2, and pose 3, it can be seen that when the test machine is in pose 3, the first-order natural frequency of the system is much smaller than that in pose 1 and pose 2; the natural frequencies of the mobile platforms are quite different in different poses.

By comparing and analyzing the natural frequencies measured by the excitation of the upper moving platform and the excitation of the lower platform in the modal experiment, it can be seen that the natural frequencies of the system measured by the two are basically the same, indicating that the excitation of the moving platform 1 and the moving platform 2 has a negative impact on the inherent frequency of the testing machine system. The frequency effect is small, which also proves the integrity of the testing machine system.

## Conclusion

The equivalent correlation coefficient solution based on the Mehler formula is efficient and accurate for nonlinear translation. In the non-Gaussian stochastic process simulation based on memoryless nonlinear translation theory, the conversion of the correlation coefficient of the Gaussian stochastic process and the correlation coefficient of the non-Gaussian stochastic process is bound to be involved, and the conversion relationship is a complex two-dimensional integral. The scope of application is limited. Starting from the transformation of the correlation coefficient, this paper introduces the Mehler formula to solve the equivalent correlation coefficient, transforms the complex two-dimensional integral into a one-dimensional numerical calculation problem, and effectively solves the problem of the scope of application. In order to study the application of flexible joints in footed robots, the dynamic differential equations of the single-leg bounce model with hip joint flexible rotation are established, and the fixed point search strategy based on the rough search of periodic cell maps and the precise search of orthogonal tables is obtained. Through a controlled study, it was found that the flexible swivel joint had a tendency to deteriorate the stability of the bouncing motion compared with the bouncing model of the free swivel hip joint under passive motion conditions. In this regard, an air-phase control method for single-leg bounce based on inverse dynamics is studied, which enables it to obtain a wider range of stable motion than the free-rotating hip bounce model. The kinematics performance of the built experimental platform is verified, and the results show that the theoretical motion trajectory and the actual motion trajectory are basically consistent in the X and Y directions, and there is a certain error in the Z direction, and the error is within the acceptable range, indicating that the experimental platform system can be used to simulate the motion of the human hip joint. The dynamic performance of the built experimental platform is verified, and the results show that the theoretical value of the driving torque calculated by the theoretical value of the dynamic parameters has a large error with the measured value, and the dynamic theoretical model cannot accurately predict the driving torque. Comprehensive analysis of the reasons shows that the driving torque error is mainly caused by the inaccurate dynamic parameters, which is of great significance to identify the actual values of the dynamic parameters. The dynamic parameter identification and verification of the built experimental platform are carried out, and the results show that the predicted value of the driving torque is in good agreement with the measured torque after the dynamic parameter identification. The confidence of the predicted value of the driving torque is increased by 10–16%, reaching about 85%, which shows that the dynamic parameter identification method in this paper has a high degree of confidence, and also verifies the accuracy of the dynamic parameter identification model and identification value.

## Data Availability

The original contributions presented in the study are included in the article/Supplementary Material, further inquiries can be directed to the corresponding author.
